# Harmonising Theory and Practice: Can Literature Supporting the Use of Music for Improved Maternal Mental Health and Mother–Infant Bonding Be Effectively Applied in an Inpatient Mother and Baby Unit?

**DOI:** 10.1192/bjo.2025.10786

**Published:** 2025-06-20

**Authors:** Alice Watts

**Affiliations:** Kent and Medway NHS and Social Care Partnership Trust, Dartford, United Kingdom

## Abstract

**Aims:** This project investigates whether mother and baby music sessions in the inpatient perinatal mental health setting can effectively reduce maternal anxiety, improve mood, and strengthen the mother–baby bond. It aims to review existing literature and reflect on the practical implementation of a pilot music group within a mother and baby unit (MBU).

MBUs are specialist inpatient wards which keep mothers united with their babies during treatment to maintain the mother–baby bond. Therapeutic activities vary across units, but music groups have been explored as a potential intervention to improve maternal mental health and bonding.

**Methods:** A literature review was conducted, identifying 8 relevant studies. The pilot music group, based on supporting literature, consisted of 6 weekly hour-long sessions, involving singing nursery rhymes and improvisation. Participants included 15 mothers with various mental health conditions. Reflections from the author/session lead and staff present were recorded to evaluate the intervention.

**Results:** Literature showed improvements to maternal anxiety and maternal mood after attending singing groups however the populations studied in most papers did not reflect those with more severe mental health conditions and few showed statistical significance. Bonding improvements were consistently noted across studies, often assessed through qualitative feedback or bonding scales. The pilot sessions were well-received, with mothers reporting relaxation and enjoyment. However, practical challenges emerged, not alluded to in previous literature, including cultural relevance of songs, emotional triggers, and varying levels of engagement amongst mothers with differing mental health conditions.

**Conclusion:** Music groups show promise as a therapeutic intervention for enhancing maternal mental health and bonding, reflected in those who attended the pilot music group. Quantitative research on its benefit in more unwell mothers admitted for treatment is still required. Challenges such as emotional triggers and the inclusion of diverse patient populations were identified in this pilot and efforts should be made to mitigate this in further interventions of a similar nature. Future research should focus on mothers with severe illness, particularly those admitted to MBUs, and explore the long-term impact of music groups as a routine intervention to support maternal–infant bonding during treatment.

